# Correction: Yamamoto, Y.; Gerbi, S.A. Development of Transformation for Genome Editing of an Emerging Model Organism. *Genes* 2022, *13*, 1108

**DOI:** 10.3390/genes14071401

**Published:** 2023-07-06

**Authors:** Yutaka Yamamoto, Susan A. Gerbi

**Affiliations:** Department of Molecular Biology, Cell Biology, and Biochemistry, Brown University Division of Biology and Medicine, Sidney Frank Hall, 185 Meeting Street, Providence, RI 02912, USA; yutaka_yamamoto@brown.edu

## Text Correction

There was a typographical error in the original publication [1]. A correction has been made to Section 2.1.1, Egg Collection and Injection, Paragraph 5, where “IE1 transactivator plasmid (pIE1/153) (0.4 ng/μL) for the injection” should be “IE1 transactivator plasmid (pIE1/153) (4 ng/μL) for the injection”. This is 1/100 of the hr5-ie1 piggyBac transposase plasmid (400 ng/μL) and not 1/1000 as erroneously published in our article.

The authors state that the scientific conclusions are unaffected. This correction was approved by the Academic Editor. The original publication has also been updated.
